# Supplementary material for: Effector–host interactome map links type III secretion systems in healthy gut microbiomes to immune modulation
Source: Nat Microbiol. 2026 Jan 26;11(2):442–60. doi: 10.1038/s41564-025-02241-y (PMC12872453; doi:10.1038/s41564-025-02241-y)
Supplement: Supplementary file 1 — Guide to Supplementary Data providing an overview of 26 thematically organized datasets, each introduced by a summary sheet describing the contents of every worksheet. [file 41564_2025_2241_MOESM1_ESM.pdf]

nature microbiology

Supplementary information https://doi.org/10.1038/s41564-025-02241-y

Effector–host interactome map links type III secretion systems in healthy gut microbiomes to immune modulation

In the format provided by the authors and unedited

Guide to Supplementary Data

The extensive supplementary data are organized in 26 Datasets that are thematically organized and described below. In every Supplementary Data, the first sheet contains a summary in which the content of every column in each of the worksheets is described.

Supplementary Data 1: T3SS identification in reference strains

This dataset includes results of T3SS prediction in 77 reference strains and the phylogenetic assignment of the 44 strains encoding complete T3SS. It includes the following tabs:

- **Table 1A** Prediction of T3SS in the 77 reference strains.
- **Table 1B** Phylogenetic assignment of 44 reference strains encoding complete T3SS.

Supplementary Data 2: TxSS identification in HBC/UHGG collections

Identification of different secretion systems using EffectiveDB, gram prediction and phylogenetic assignment for 4,752 genomes from HBC and UHGG.

Supplementary Data 3: T3SS identification in metagenome assemblies

This dataset includes results of T3SS prediction across 16,179 Pseudomonadota metagenome assemblies, species-level phylogenetic assignment of 770 MAGs encoding complete T3SS. It includes the following tabs:

- **Table 3A** T3SS in commensal microbiota using metagenome datasets.
- **Table 3B** Phylogenetic assignments for the 770 MAGs encoding complete T3SS.
- **Table 3C** Genera, species names, and genomes encoding complete T3SS from the samples of three genome sets (data related to figure 1b).

Supplementary Data 4: Prediction of T3SS effectors

This dataset includes results of predicting 3,002 effectors in 44 reference strains and 182 effectors in 770 T3SS-positive MAGs using three complementary machine-learning models. It includes the following tabs:

- **Table 4A** Prediction of 3002 effectors in known 44 reference strains
- **Table 4B** Prediction of 182 effectors in 770 T3SS+ MAGs

Supplementary Data 5: Commensal versus pathogen effectors

This dataset includes sequence-level comparisons between commensal or MAG T3SS effectors and known pathogen effectors, together with Pfam domain annotations. It also provides a comparative summary of Pfam domains enriched in commensal effectors and their occurrence. It includes the following tabs:

- **Table 5A** Sequence similarity between commensal strain effectors and pathogenic effectors.
- **Table 5B** Sequence similarity between MAG effectors and pathogenic effectors.
- **Table 5C** Pfam domain annotation of effector proteins from commensal strains and metagenomes
- **Table 5D** Pfam domain annotation of effector proteins from human and vertebrate pathogens
- **Table 5E** Pfam domain annotation of reviewed human proteins from the UniProtKB/Swiss-Prot reference proteome
- **Table 5F** Comparative summary of significantly enriched Pfam domains in commensal effectors and their occurrence in pathogen and human proteins

Supplementary Data 6: Structural effector analysis and Foldseek clustering

Structural effector analysis and Foldseek clustering. This dataset presents structural mapping and clustering analyses of effectors based on AlphaFold models and Foldseek clustering. It includes mappings of effector IDs to their corresponding AlphaFold model IDs, Foldseek clustering results at different bidirectional query coverages and *E* values, classification of cloned effectors by cluster type across Foldseek runs, and results from permutation tests assessing cluster type distributions for all, vertebrate, and human pathogen effectors. It includes the following tabs:

- **Table 6A** Effector AlphaFold mapping
- **Table 6B** Foldseek cluster output (coverage = 0.5, e-value = 0.01)
- **Table 6C** Foldseek cluster output (coverage = 0.7, e-value = 0.01)
- **Table 6D** Foldseek cluster output (coverage = 0.9, e-value = 0.01)
- **Table 6E** Cloned effector structure cluster type
- **Table 6F** Proportion of cluster types for Foldseek cluster runs
- **Table 6G** The proportion of each cluster type for all Foldseek cluster permutation tests (n=10,000), all pathogen effectors considered

- **Table 6H** Permutation test *P* values for cluster type proportion distributions, all pathogen effectors considered
- **Table 6I** The proportion of each cluster type for all Foldseek cluster permutation tests (n = 10,000), only vertebrate effectors considered
- **Table 6J** Permutation test P values for cluster type proportion distributions, only vertebrate effectors considered
- **Table 6K** The proportion of each cluster type for all Foldseek cluster permutation tests (n = 10,000), only human effectors considered
- **Table 6L** Permutation test P values for cluster type proportion distributions, only human effectors considered

Supplementary Data 7: Effector cloning

Effector cloning. This dataset presents information related to the cloning of effectors. It includes primers used for cloning, effectors from selected strains, cloned effector ORFs (Gateway entry clones, HuMEOme_v1), and primers used for cloning candidate effectors into pLac_FL_HiBit C-terminus tag in mammalian cells for the injection assay. It includes the following tabs:

- **Table 7A** Cloning primers
- **Table 7B** Cloned effector ORFs (Gateway entry clones) (HuMEOme_v1)
- **Table 7C** Cloning primers for injection assay

Supplementary Data 8: Species GTDB taxonomy IDs and abbreviations used in this study

Phylogenetic assignments of strains used in this study. This dataset presents a summary of the phylogenetic assignments of all strains included in this study based on GTDB taxonomy, together with their abbreviations and catalog numbers.

Supplementary Data 9: Effector identifiers and abbreviations used in this study

Effector identifiers and abbreviations used in this study. This dataset presents a reference table listing all effectors analyzed in the study, including their sequence IDs, protein definitions, strain abbreviations, and effector short names.

Supplementary Data 10: Injection assays raw data and statistics

Injection assays raw data and statistics. This dataset presents raw and summarized luminescence data from effector injection assays performed with Salmonella Typhimurium and *Edwardsiella tarda* effectors relative to the *Salmonella* SipA control. It includes the following tabs:

- **Table 10A** Raw luminescence values from injection assay with *S.* Typhimurium strains
- **Table 10B** Summary data of the injection assay with *S.* Typhimurium strains
- **Table 10C** Raw luminescence values from injection assay with *Edwardsiella tarda*
- **Table 10D** Summary data of the injection assay with *Edwardsiella tarda*

Supplementary Data 11: Human-microbiome meta-interactome

This dataset presents protein-protein interactions between microbiome effectors and human proteins. It includes host-effector interactions identified by yeast-two-hybrid (Y2H) assays, effector homology groups based on sequence similarity, and degree distributions of human and effector proteins within $HuMMI_{MAIN}$, together with a summary of human proteins targeted by bacterial effectors and convergence analysis results. It includes the following tabs:

- **Table 11A** Total list of identified protein-protein interactions between effector and human proteins.
- **Table 11B** Effector homology groups
- **Table 11C** Human effector-degree distribution of $HUMMI_{MAIN}$
- **Table 11D** Effector human-degree distribution of $HUMMI_{MAIN}$
- **Table 11E** Target summary and convergence results
- **Table 11F** Well-specific primers
- **Table 11G** Plate-specific primers

Supplementary Data 12: Reference sets

This dataset presents curated reference sets used for benchmarking effector-human protein interactions. It includes list of previously identified binary interactions between bacterial pathogenic effectors and human proteins (bhLit-BM-v1) and a negative control reference set of random bacterial and human protein pairs (bhRRS-v1). It includes the following tabs:

- **Table 12A** A curated list of previously identified binary interactions between bacterial pathogenic effectors and human proteins (bhLit-BM-v1).
- **Table 12B** A negative control reference set of random bacterial and human protein pairs (bhRRS-v1)

Supplementary Data 13: Assay sensitivity

This dataset presents yeast-two-hybrid (Y2H) assay data assessing sensitivity using positive and negative reference sets. It includes yeast growth scoring, identification of interaction protein pairs, and Y2H scoring summaries for bhLit-BM-v1, bhRRS-v1, hsPRS-v2, and hs-RRS-v2 tested in two configurations. It includes the following tabs:

- **Table 13A** Yeast growth scoring and identification of interacting pairs for bhLit-BM-v1 and bhRRS-v1 by Y2H.
- **Table 13B** Y2H scoring summary for bhLit-BM-v1 and bhRRS-v1
- **Table 13C** Yeast growth scoring and identification of interacting pairs for hsPRS-v2 and hsRRS-v2 in configuration 1.
- **Table 13D** Yeast growth scoring and identification of interacting pairs for hsPRS-v2 and hsRRS-v2 in configuration 2.
- **Table 13E** Y2H scoring summary for hsPRS-v2 and hsRRS-v2

Supplementary Data 14: Validation rate

This dataset presents raw and summarized data from yN2H validation assays. It includes raw luminescence (RLU) measurements, the number of hits above threshold, total pair configurations tested, and statistical tests comparing validation rates between protein pair sets using Fisher's exact test. It includes the following tabs:

- **Table 14A** Raw data yN2H
- **Table 14B** yN2H result summary

Supplementary Data 15: Interactions between pathogen effectors and human proteins downloaded from IMEx

This dataset presents interaction between pathogens effectors and human targets proteins curated from IMEx consortium database. The table presents UniProt identifiers for effectors and human proteins, gene symbol, taxonomic identifiers, interaction types, detection methods, and supporting PubMed references.

Supplementary Data 16: The relationship between effector interaction profile and effector sequence similarity

This dataset presents pairwise of effector-effector interaction profile similarity using Jaccard index, based on sequence similarity among effectors.

Supplementary Data 17: AlphaFold2 interaction interface in HuMMI

This dataset presents AlphaFold2-based structural predictions and comparative analyses of HuMMI interaction interfaces. It includes confident interface models with at least five residue-residue contacts, assessment of interface similarity among effectors and human proteins at different PAE cutoffs, and domain annotations overlapping with models HuMMI interaction interfaces. It includes the following tabs:

- **Table 17A** Prediction of interaction interface structure by AlphaFold2
- **Table 17B** Human interface structure similarity
- **Table 17C** Effector interface structure similarity
- **Table 17D** Interface domain annotation

Supplementary Data 18: Domain-motif interface predictions

This dataset presents the predicted interaction interfaces inferred by mimicINT, including both domain-motif and domain-domain interaction interfaces. It includes the following tabs:

- **Table 18A** Domain-motif interaction interfaces
- **Table 18B** Domain-domain interaction interfaces

Supplementary Data 19: Holdup assay and validation of PBM-PDZ interface predictions

This dataset presents results from holdup (HU) assays validating predicted PBM-PDZ interaction interfaces from HuMMI. It includes raw and processed HU assays data together with validation of domain interface predictions and their comparison to Y2H. It includes the following tabs:

- **Table 19A** Raw and treated data holdup runs
- **Table 19B** Validation of domain interface identification by holdup assay

Supplementary Data 20: Functional enrichment analysis of targets

This dataset presents results of functional and metabolic pathway enrichment analyses of human proteins targeted by effectors and pathogens.

- **Table 20A** Functional enrichment analysis of effector targets
- **Table 20B** Functional enrichment analysis of effector targets that are subject to convergence
- **Table 20C** Metabolic pathways enrichment analysis for effector targets
- **Table 20D** Functional enrichment analysis of pathogen targets (n=217).

Supplementary Data 21: Genetic predisposition enrichment of effector targets

This dataset presents results of enrichment analyses testing effector targets for overlap with trait-associated genes from the OpenTargets genetics portal using Fisher's exact test with HuRI proteins as background. Reported values include experimental factor ontology (EFO) identifiers and trait labels, numbers of causal genes in HuRI, intersecting effector targets, odds ratios, P values, and FDR-corrected significance (data related to **Fig. 4b**).

Supplementary Data 22: The prevalence of HuMMI effector proteins was determined across the OhJ_2014 cohort of 285 skin samples

This dataset presents the prevalence of HuMMI effectors detected in metagenomes from 285 human skin samples. Reported values include effector abbreviations, the percentage of positive samples, and the number of samples in which each effector was detected.

Supplementary Data 23: Genetic predisposition trait enrichment in effector target neighborhoods identified by RWR

This dataset present results of Random Walk with Restart (RWR) analyses assessing enrichment of genetic predisposition traits with HuRI-derive neighborhoods of commensal effector targets. It includes trait-level enrichments, their categorization by EFO disease classes, and immunological EFO term enriched in effector-target neighborhoods, with closely related traits consolidated. It includes the following tabs:

- **Table 23A** Genetic predisposition trait enrichment across HuRI-derived neighborhoods of commensal effectors using Random Walk with Restart (RWR).
- **Table 23B** Disease-category attributes of genetic predisposition trait associations in effector target network neighborhoods.
- **Table 23C** Immunological genetic predisposition traits (EFO terms) enriched in effector target neighborhoods.

Supplementary Data 24: NF-kB assay

This dataset compiles luciferase measurements, F/R values, and statistical results from NF-kB reporter assays performed on unstimulated and TNF-stimulated cells, together with summary data for the empty vector used in these assays.

- **Table 24A** NF-kB reporter assay on untreated cells
- **Table 24B** NF-kB reporter assay on treated cells
- **Table 24C** Summary data of the empty vector used in the NF-kB reporter assay

Supplementary Data 25: Cytokine assay

This dataset includes cytokine concentrations from Caco2 cell assays expressing effector Cpa-12, baseline summaries per cytokine, and statistical analyses using Kruskal-Wallis with Dunn's test (data related to Fig. 5c-d and Extended Data Fig. 4d

- **Table 25A** Cytokine concentrations per sample for effector Cpa_12 assays in pg/mL calculated based on baseline.
- **Table 25B** Baseline QC for cytokine.
- **Table 25C** Statistic for cytokine analysis of Cpa_12.
- **Table 25D** Cytokine concentrations per sample for bacterial effectors assays
- **Table 25E** Statistics for cytokine analysis.

Supplementary Data 26: The prevalence of HuMMI effector proteins in IBD versus healthy patients

This dataset presents the prevalence of HuMMI effectors detected in metagenomes from patients with Crohn's disease (CD) and ulcerative colitis (UC) compared to non-IBD controls (data related to **Fig. 5e, 5f**, and **5g**).
