# Supplementary material for: Effector–host interactome map links type III secretion systems in healthy gut microbiomes to immune modulation
Source: Nat Microbiol. 2026 Jan 26;11(2):442–60. doi: 10.1038/s41564-025-02241-y (PMC12872453; doi:10.1038/s41564-025-02241-y)
Supplement: Supplementary file 30 — Unprocessed western blots. [file 41564_2025_2241_MOESM30_ESM.pdf]

Source Data of Extended Data Figure 1f

Bands of samples with a green border are shown in Extended Figure 1f.

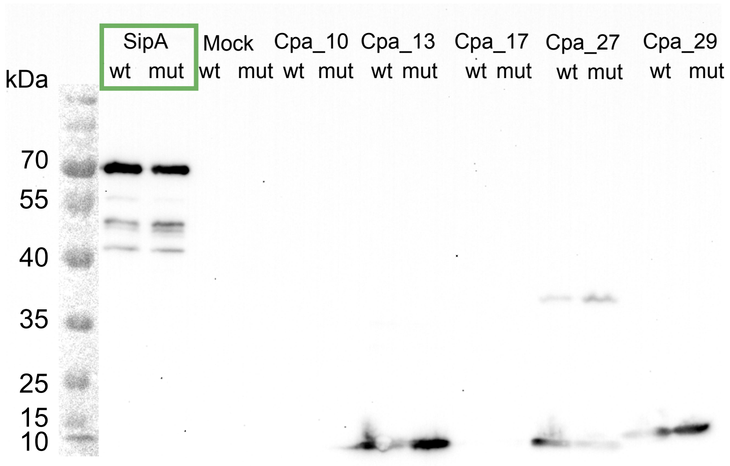

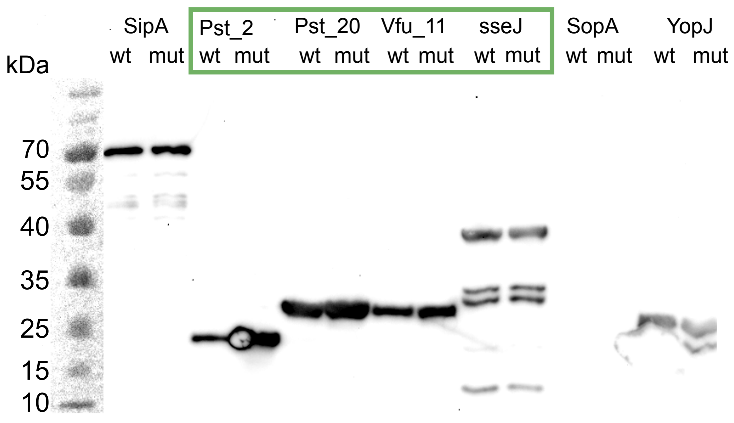

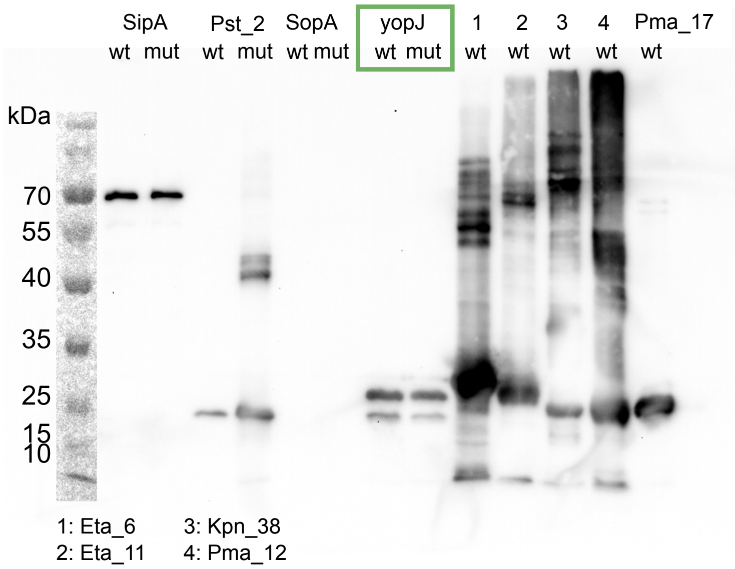

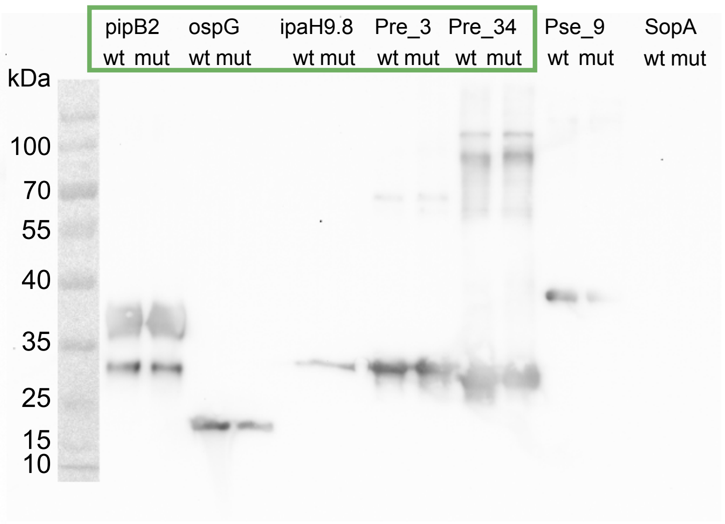

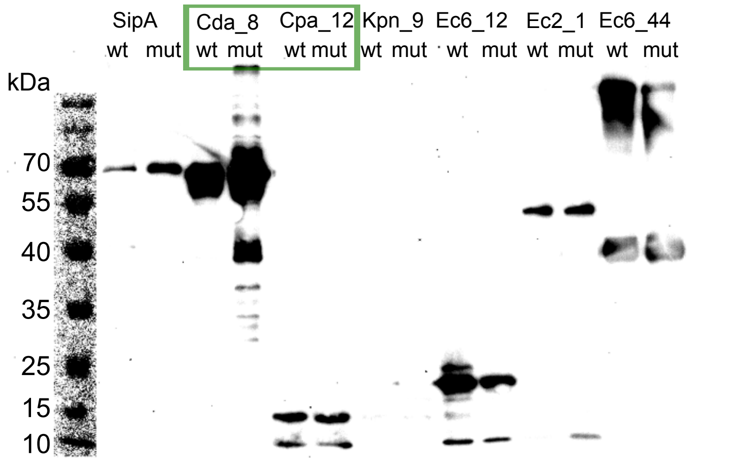

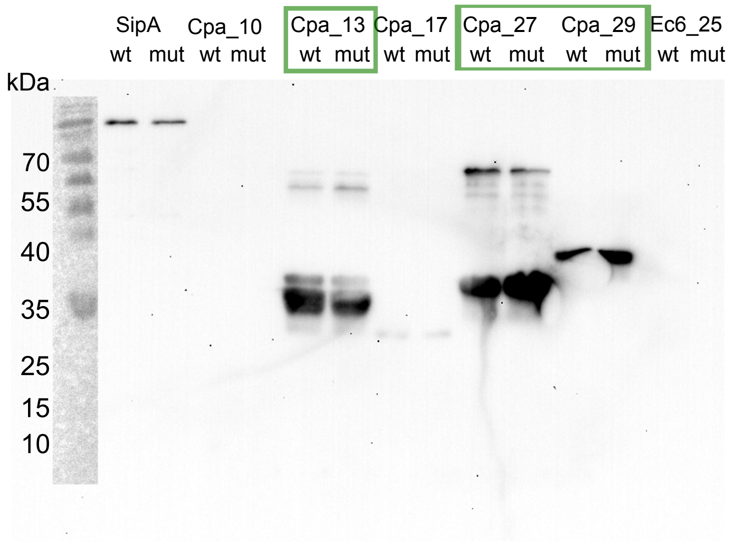

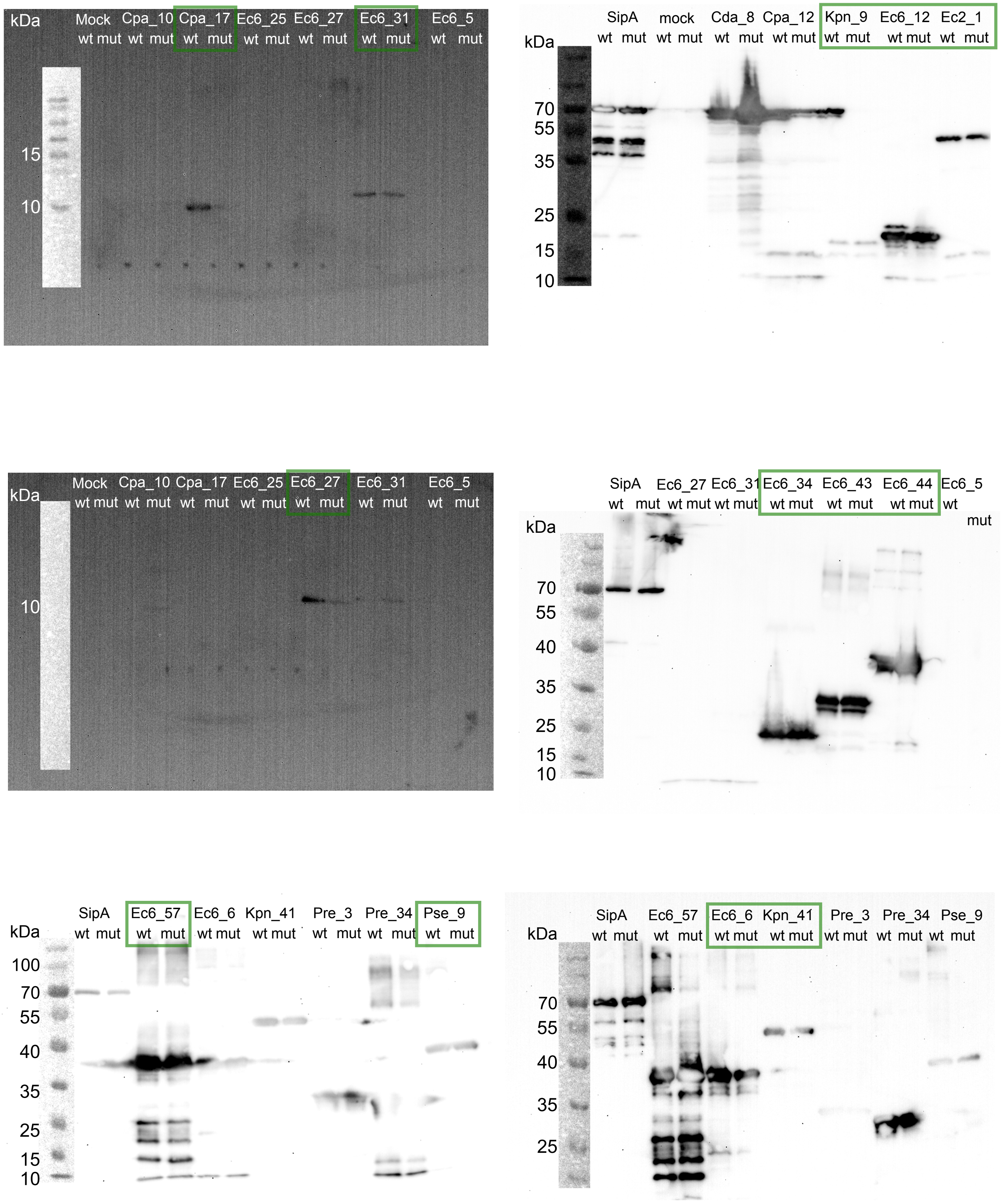
kDa
Mock Cpa_10 Cpa_17 Ec6_25 Ec6_27 Ec6_31 Ec6_5
wt mut wt mut wt mut wt mut wt mut wt mut wt mut
15
10
SipA mock Cda_8 Cpa_12 Kpn_9 Ec6_12 Ec2_1
kDa
wt mut wt mut wt mut wt mut wt mut wt mut wt mut
70
55
35
25
15
10
Mock Cpa_10 Cpa_17 Ec6_25 Ec6_27 Ec6_31 Ec6_5
kDa
wt mut wt mut wt mut wt mut wt mut wt mut wt mut
10
SipA Ec6_27 Ec6_31 Ec6_34 Ec6_43 Ec6_44 Ec6_5
wt mut wt mut wt mut wt mut wt mut wt mut wt
mut
kDa
70
55
40
35
25
15
10
SipA Ec6_57 Ec6_6 Kpn_41 Pre_3 Pre_34 Pse_9
kDa
wt mut wt mut wt mut wt mut wt mut wt mut wt mut
100
70
55
40
35
25
15
10
SipA Ec6_57 Ec6_6 Kpn_41 Pre_3 Pre_34 Pse_9
kDa
wt mut wt mut wt mut wt mut wt mut wt mut wt mut
70
55
40
35
25

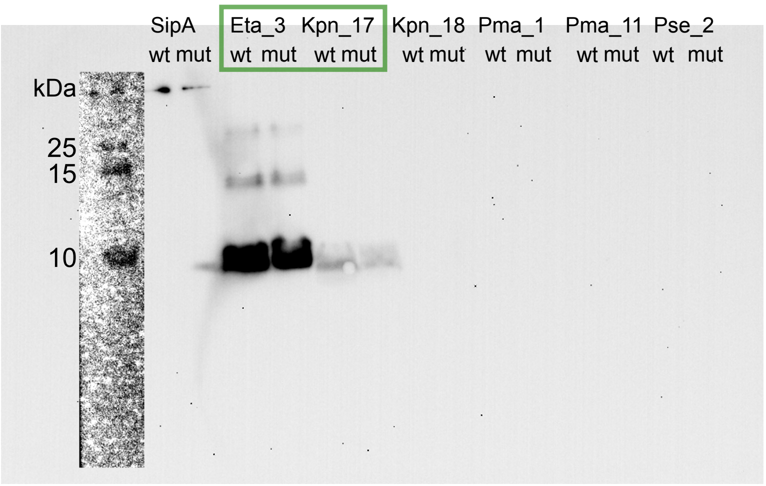
kDa
25
15
10
SipA
wt mut
Eta_3
wt mut
Kpn_17
wt mut
Kpn_18
wt mut
Pma_1
wt mut
Pma_11
wt mut
Pse_2
wt mut

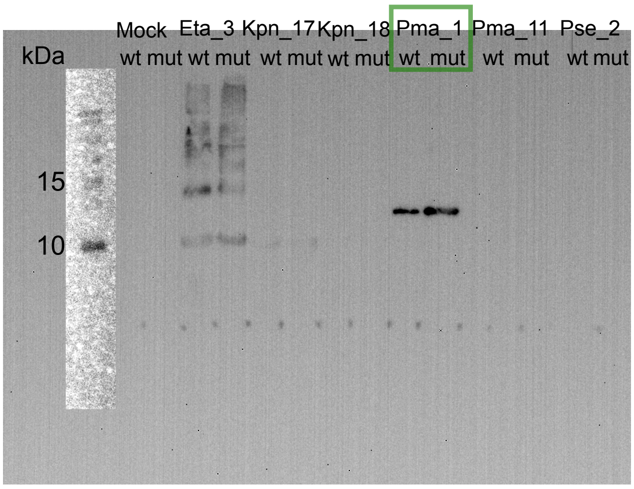
kDa
15
10
Mock
wt mut
Eta_3
wt mut
Kpn_17
wt mut
Kpn_18
wt mut
Pma_1
wt mut
Pma_11
wt mut
Pse_2
wt mut

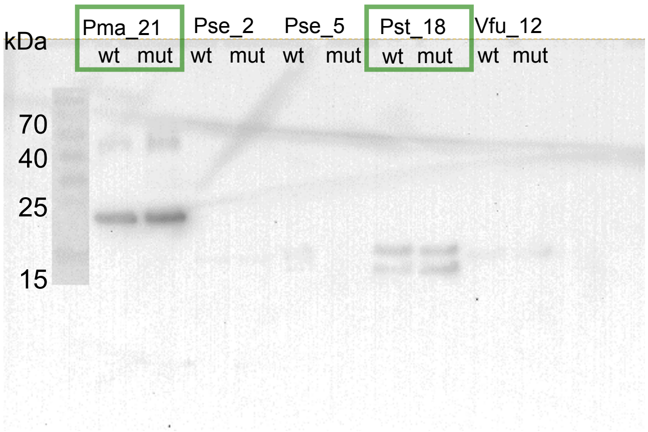
kDa
70
40
25
15
Pma_21
wt mut
Pse_2
wt mut
Pse_5
wt mut
Pst_18
wt mut
Vfu_12
wt mut
