# Supplementary material for: Effector–host interactome map links type III secretion systems in healthy gut microbiomes to immune modulation
Source: Nat Microbiol. 2026 Jan 26;11(2):442–60. doi: 10.1038/s41564-025-02241-y (PMC12872453; doi:10.1038/s41564-025-02241-y)
Supplement: Supplementary file 31 — Unprocessed western blots. [file 41564_2025_2241_MOESM31_ESM.pdf]

Source Data of Extended Data Figure 2c

Bands of samples with a green border are shown in Extended Figure 2c.

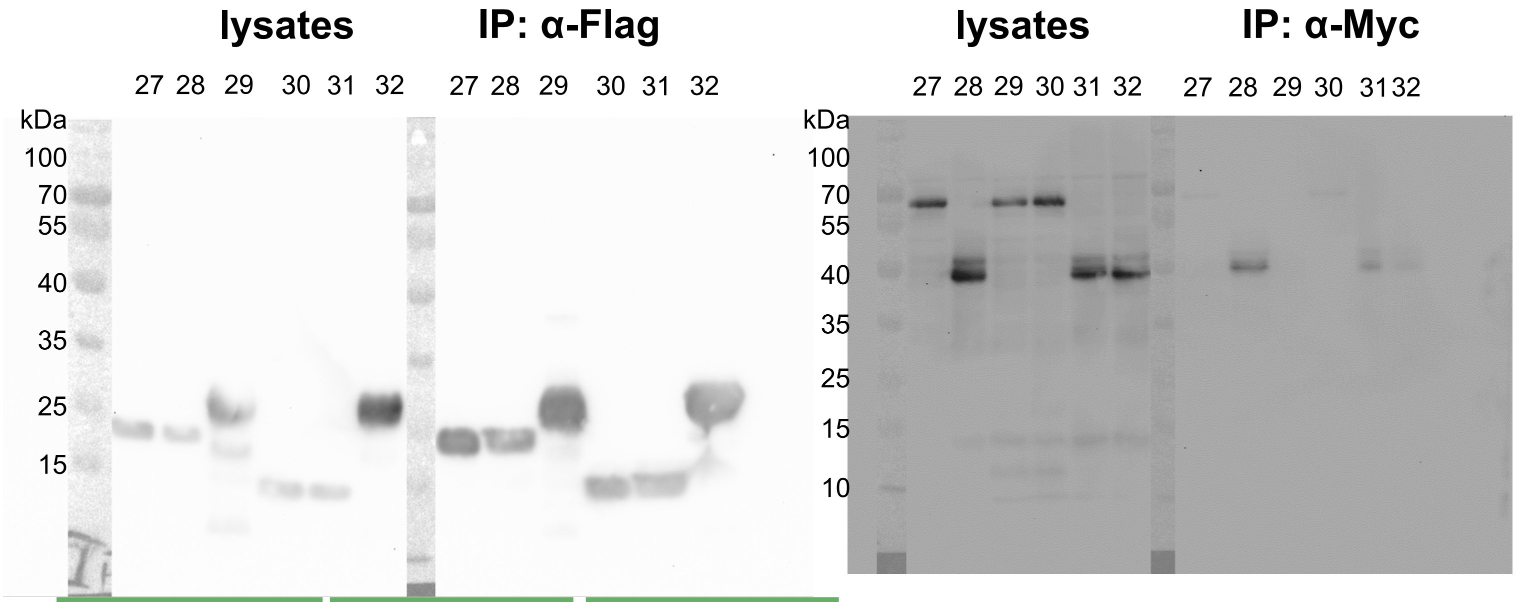

27: Efe_11 - REL
28: Efe_11 - TRAF1
29: GFP - REL
30: Kpn_3 - REL
31: Kpn_3 - TRAF1
32: GFP - TRAF1

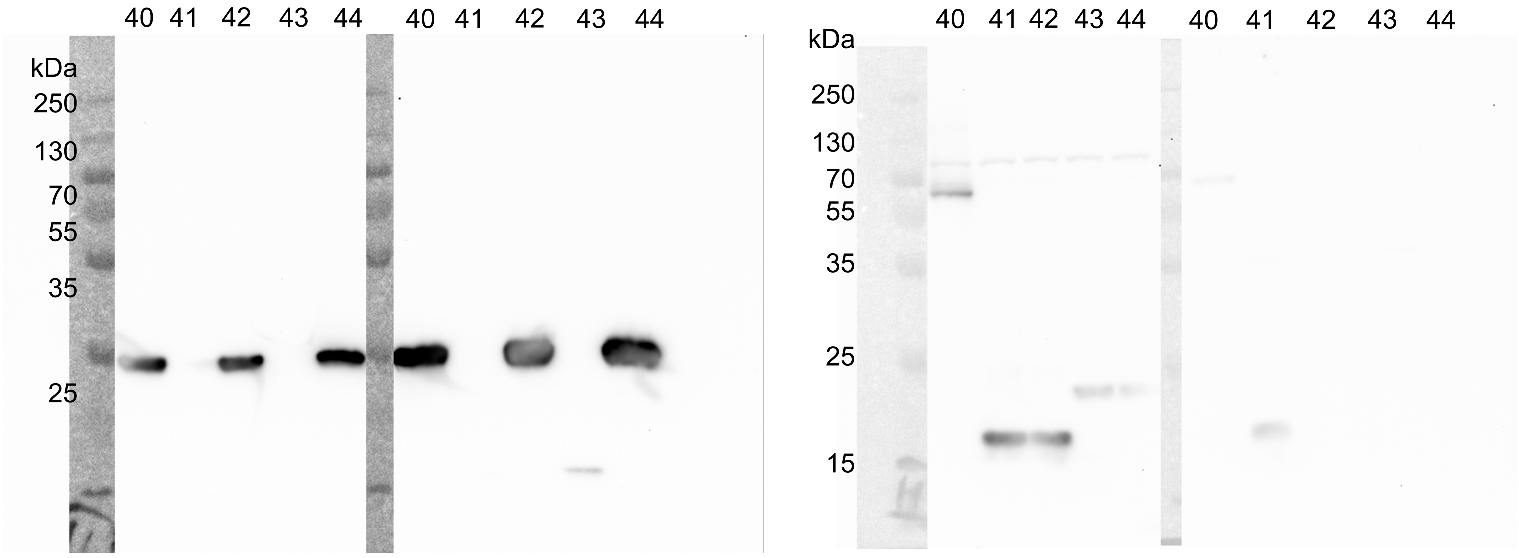

40: GFP - MID1
41: Mmo_5 - TBD52L1
42: GFP - TBD52L1
43: Vfu_1 - CNOT7
44: GFP - CNOT7

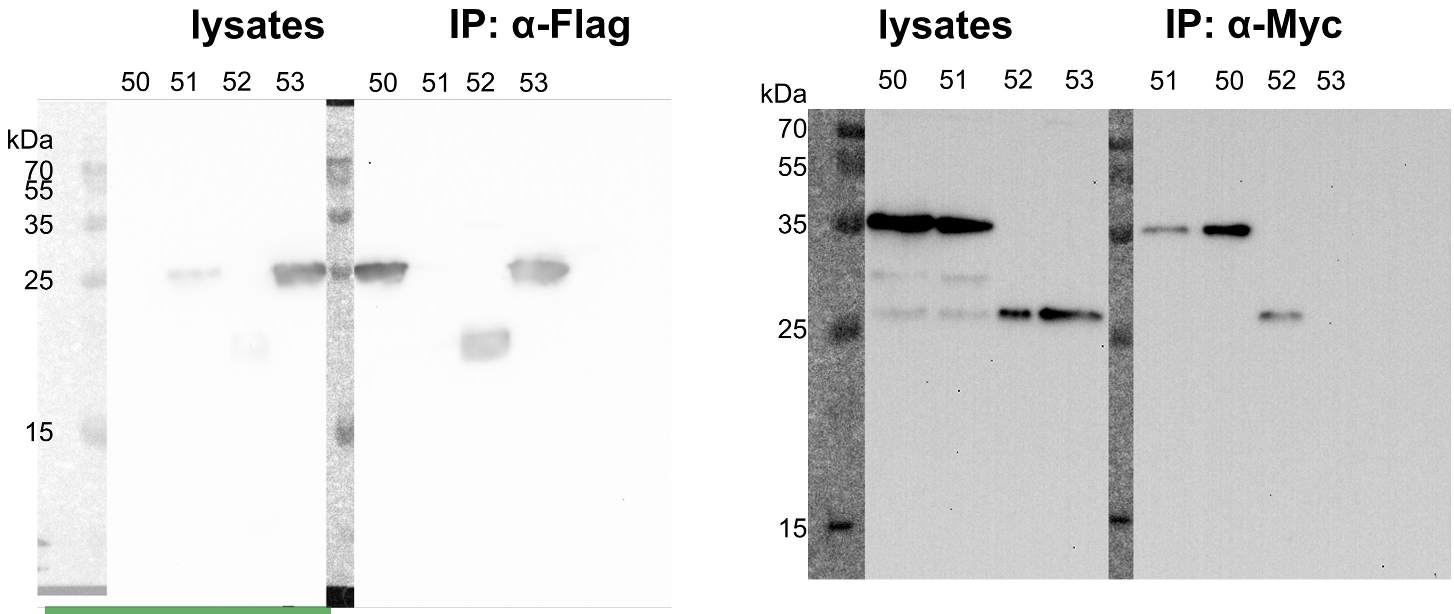

50: Kpn_5 - MAGEA8
51: GFP - MAGEA8
52: Pfa_4 - NOTO
53: GFP - NOTO

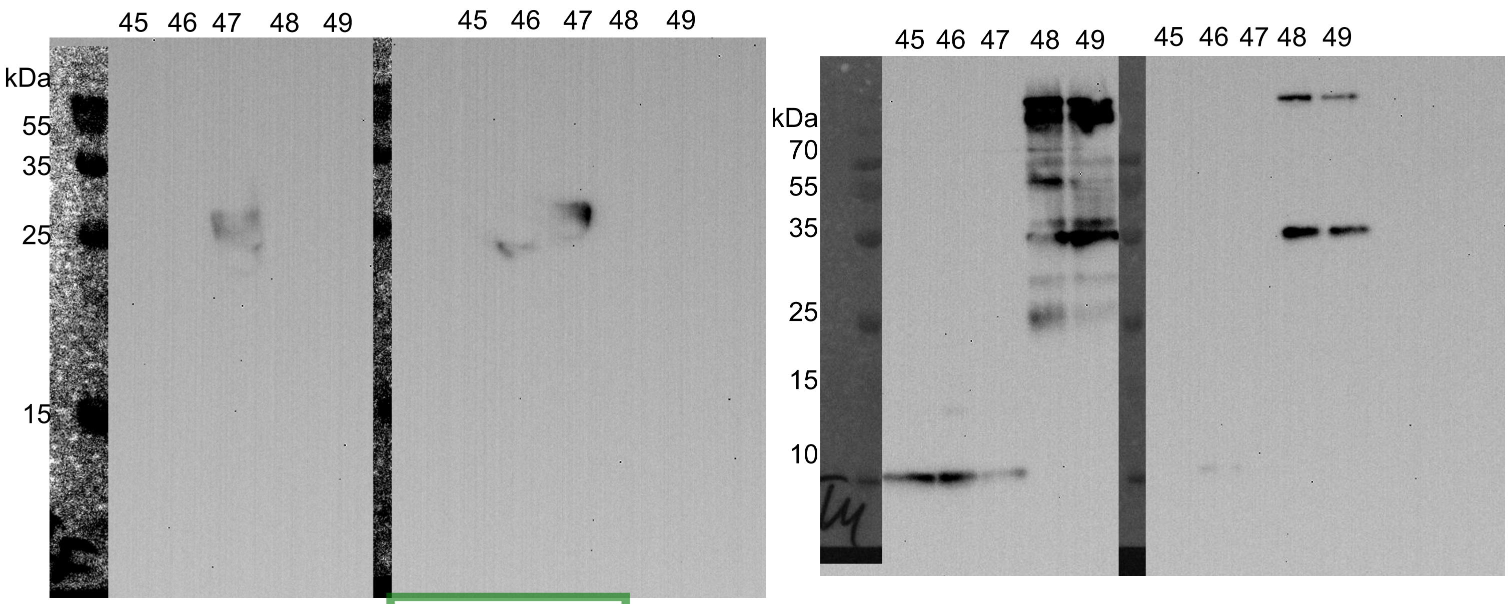

45: Ec6_20 - DYNLT1
46: Cpa_10 - DYNLT1
47: GFP - DYNLT1
48: Cpa_1 - MAGEA6
49: GFP - MAGEA6

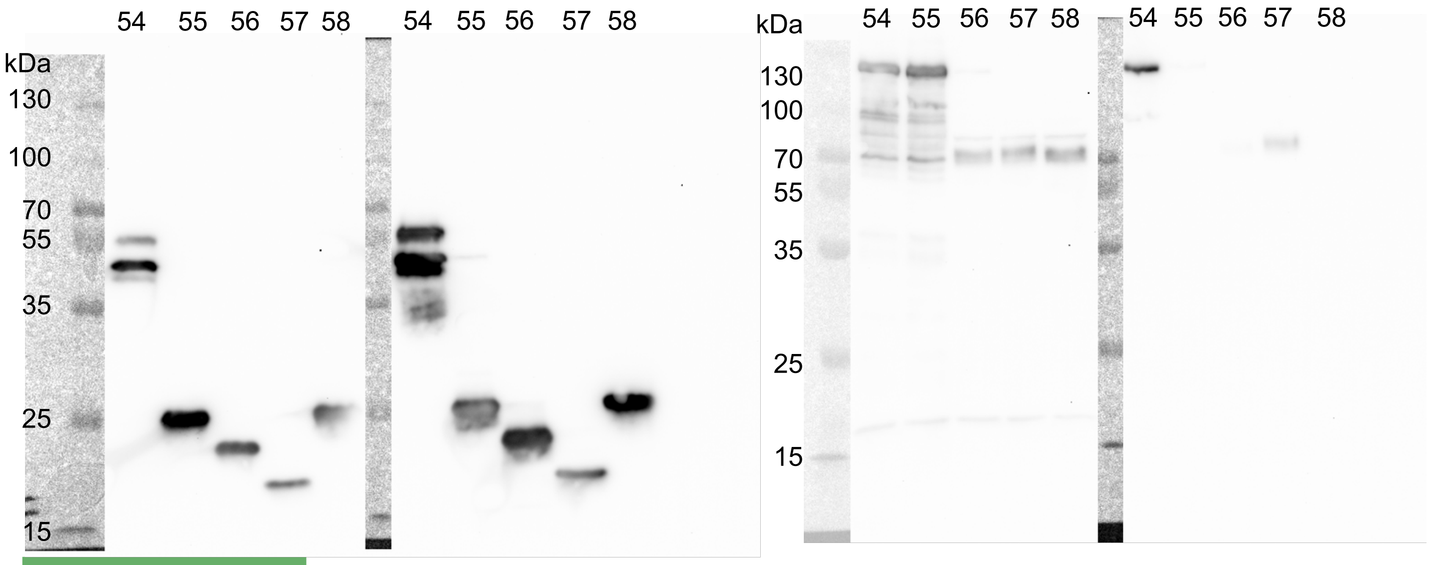

54: met_26 - MAGI1
55: GFP - MAGI1
56: Efe_11 - TCF4
57: Kpn_3 - TCF4
58: GFP - TCF4

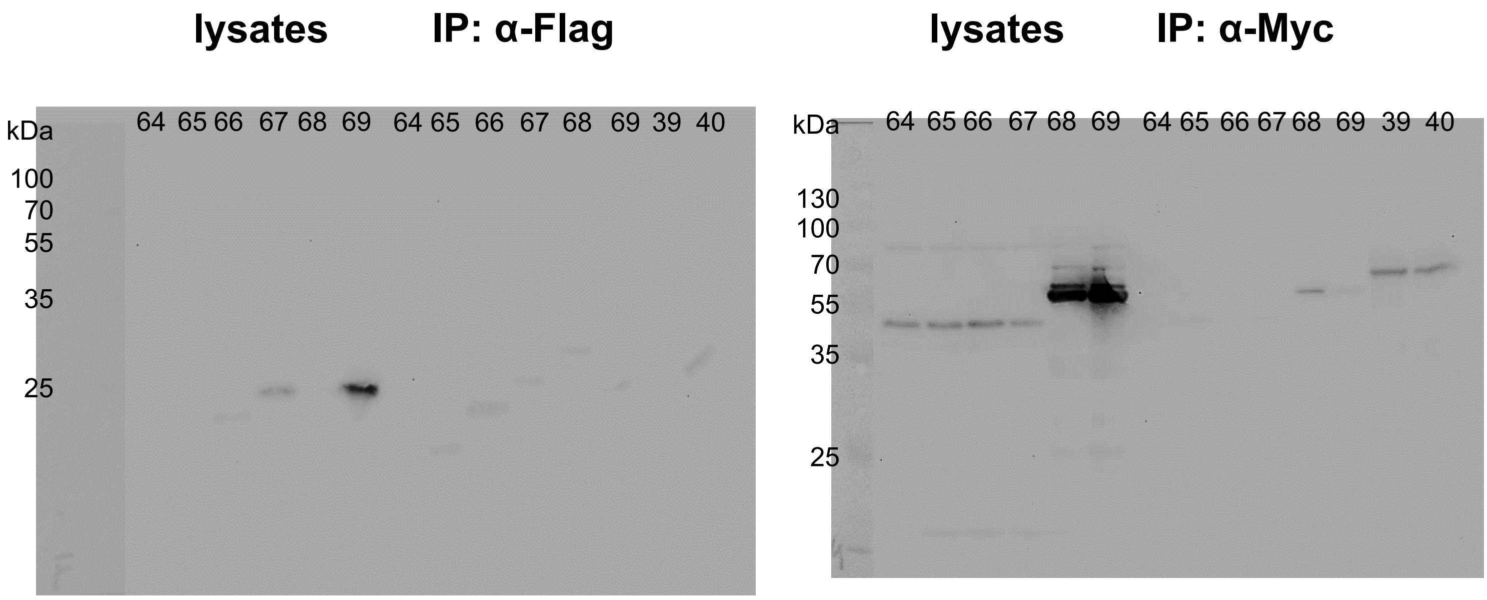

64: Cyo_12 - TRAF2
65: Kpn_3 - TRAF2
66: Efe_11 - TRAF2
67: GFP - TRAF2
68: Ec6_12 - UBQLN1
69: GFP - UBQLN1
39: met_7 - MID1
40: GFP - MID1
