# Supplementary material for: Effector–host interactome map links type III secretion systems in healthy gut microbiomes to immune modulation
Source: Nat Microbiol. 2026 Jan 26;11(2):442–60. doi: 10.1038/s41564-025-02241-y (PMC12872453; doi:10.1038/s41564-025-02241-y)
Supplement: Supplementary file 32 — Unprocessed western blots. [file 41564_2025_2241_MOESM32_ESM.pdf]

Source Data of Figure 2e

Bands of samples with a green border are shown in Figure 2e.

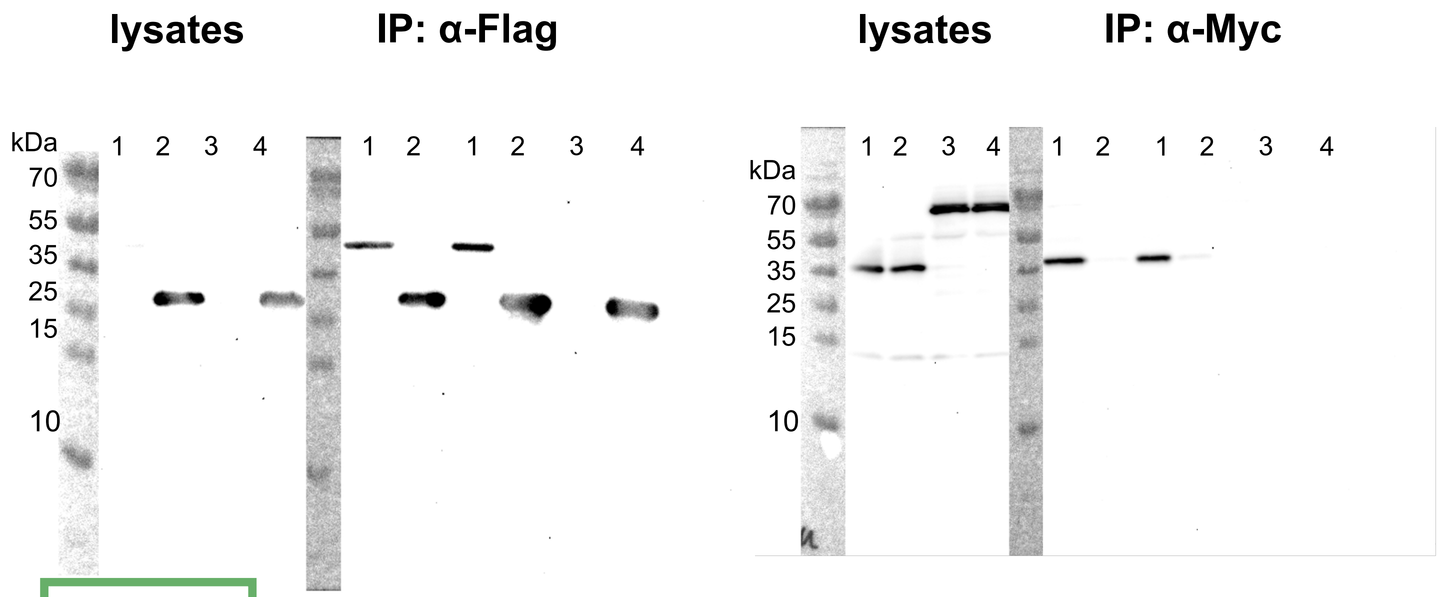

1: espG - NMI
2: GFP - NMI

3: Ec6_12 - UBQLN1
4: GFP - UBQLN1

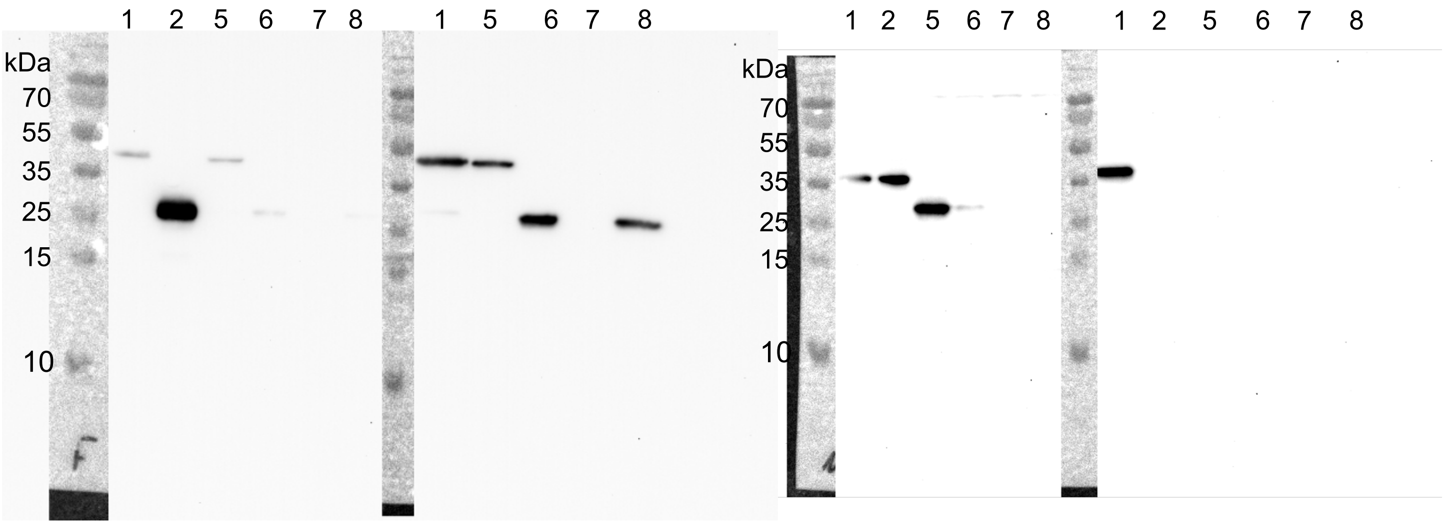

1: espG - NMI
2: GFP - NMI

5: Ec6_6 - EIF2B1
6: GFP - EIF2B1

7: Pre_21 - PPAR13B
8: GFP - PPAR13B

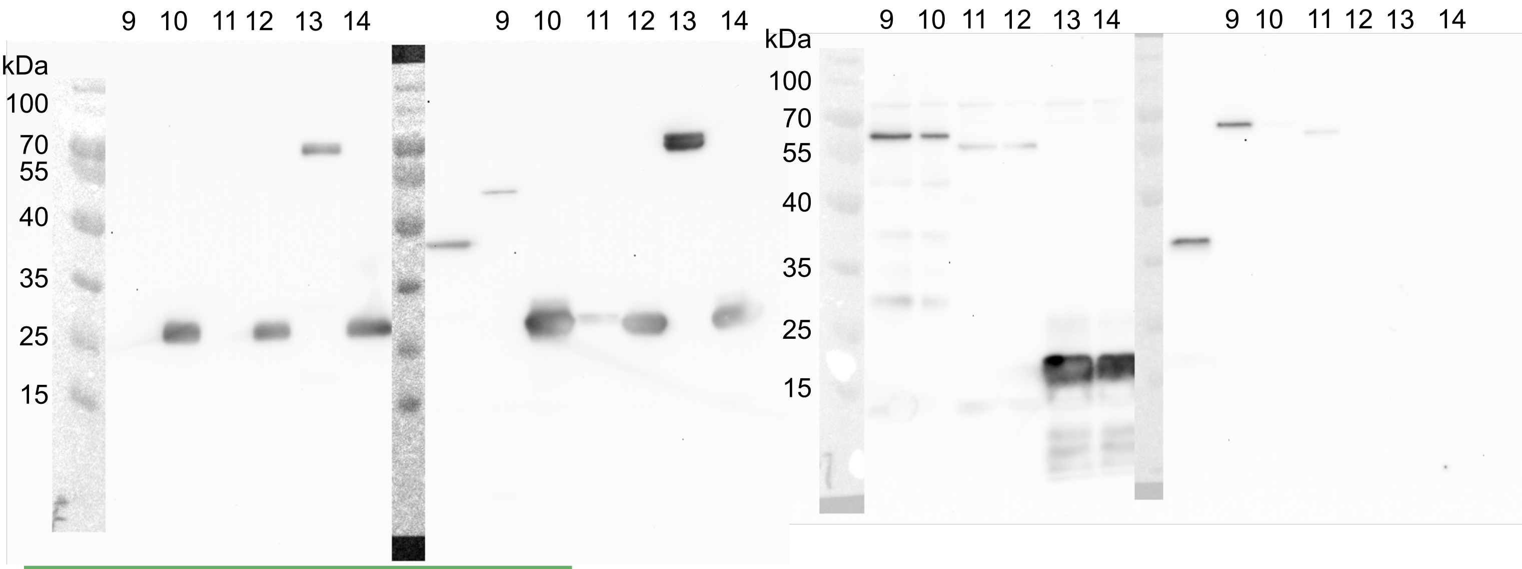

9: Efe_13 - NAP1L2
10: GFP - NAP1L2

11: Efe_14 - MPP7
12: GFP - MPP7

13: B7UM99 - HPCAL1
14: GFP - HPCAL1

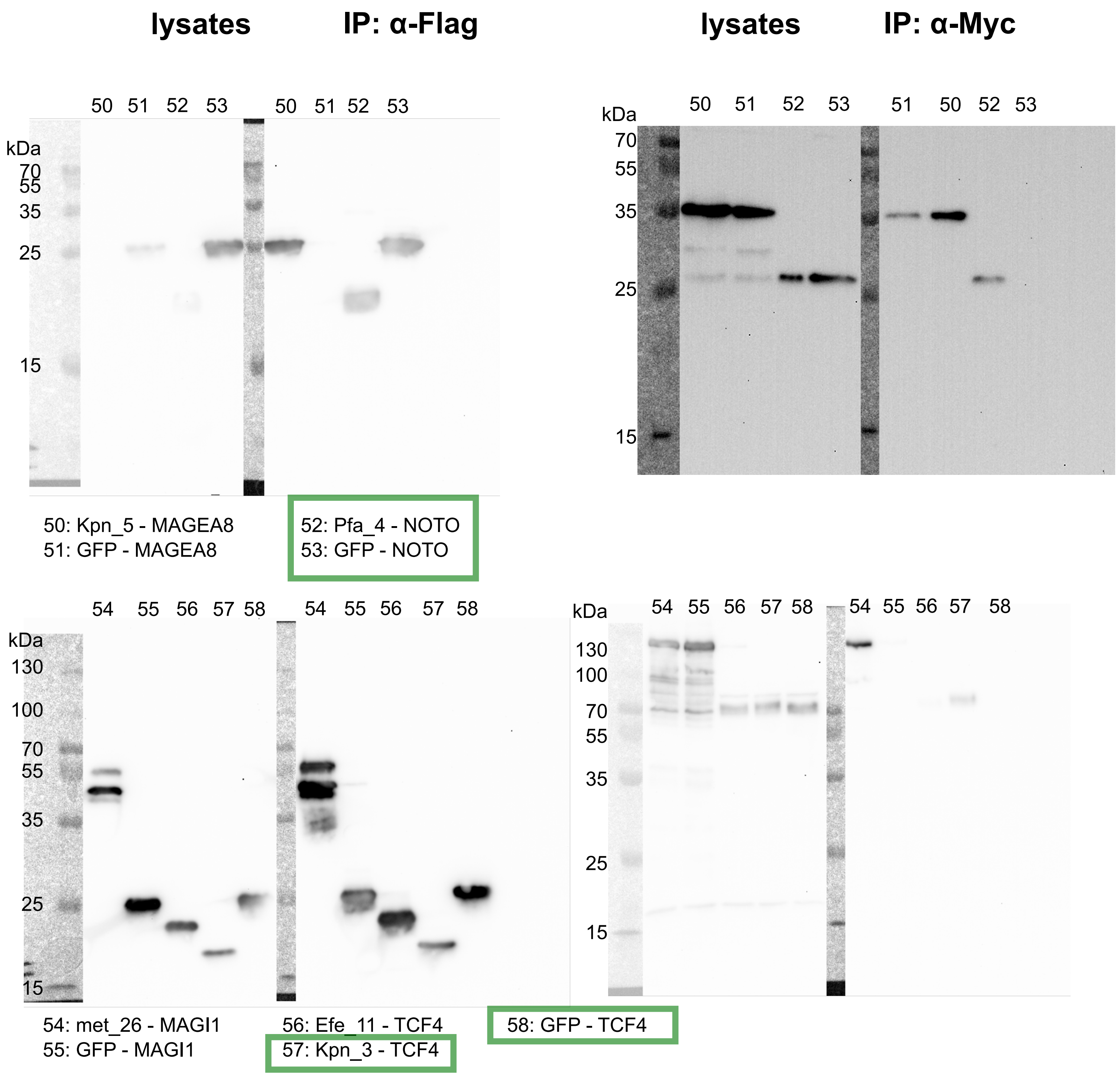
lysates
IP: α-Flag
lysates
IP: α-Myc
50 51 52 53 50 51 52 53
kDa
70
55
35
25
15
50 51 52 53 51 50 52 53
kDa
70
55
35
25
15
50: Kpn_5 - MAGEA8
51: GFP - MAGEA8
52: Pfa_4 - NOTO
53: GFP - NOTO
54 55 56 57 58 54 55 56 57 58
kDa
130
100
70
55
35
25
15
kDa
54 55 56 57 58 54 55 56 57 58
130
100
70
55
35
25
15
54: met_26 - MAGI1
55: GFP - MAGI1
56: Efe_11 - TCF4
57: Kpn_3 - TCF4
58: GFP - TCF4
